# The contribution of the foreign population to the high level of infant mortality in Switzerland: a demographic analysis

**DOI:** 10.1186/s12884-017-1332-6

**Published:** 2017-05-25

**Authors:** Philippe Wanner, Paola Bollini

**Affiliations:** Institute of Demography and Socioeconomics, University of Geneva, Bd du Pont d’Arve, 1211 Genève 4, Switzerland

**Keywords:** Infant Mortality, Reproductive health, Migrant health

## Abstract

**Background:**

In 2011 Switzerland reported the highest infant mortality rate among Western European countries, as well as the highest percentage of foreign population (23%). The comparison of the Swiss and foreign population in terms of reproductive health has received so far insufficient attention. The aim of the present study is to analyze the infant (IMR) and neonatal mortality rates (NMR) of Swiss and foreign children over the last 30 years.

**Methods:**

Vital statistics from the period 1980 to 2011 were used to compute IMR and NMR according to year and/or citizenship. The main analyses were made contrasting Swiss versus foreigners as a single category, as well as by country of origin. Comparisons between groups were done using relative risks.

**Results:**

In 1980–1989, IMR was 14% higher among foreign children as compared to Swiss children, and NMR 28% higher. In 2006–2010, IMR was 18% higher among foreign children than among Swiss children, and NMR 29% higher. The highest gap of IMR was observed during the period 1990–1993 (+21%). Looking at single countries, in 2008–2010 children of migrants from Germany, Portugal, Turkey, Italy, France, Kosovo and Spain had a higher level of IMR as compared to Swiss children.

**Conclusions:**

The analysis of vital statistics confirms that over the last 30 years the gap of IMR and NMR between Swiss and foreign children has not decreased. Whatever the combination of mechanisms, which cause the observed difference, this fundamental inequity needs to be investigated and remedied by a large scale, concerted effort.

## Background

In 2011, Switzerland reported the highest infant mortality rate (IMR) among Western European countries [1]. In the same year, Swiss IMR (3.8 per 1,000) was 81% higher than in Sweden (2.1 per 1,000) and 5% to 15% higher than in neighboring countries (Italy 3.4 per 1,000, France 3.5 per 1,000, Austria and Germany 3.6 per 1000). This relatively high level of infant mortality is a paradox, as Switzerland performs well in other health and social indicators as compared to neighboring countries: for instance, it had the highest life expectancy among industrialized countries in 2011 (82.8 years for both sexes as compared to an average of 80.1 years for industrialized countries), good access to care (measured for instance by high health care coverage and low level of inequalities in access to consultations) and a high proportion of health care expenditure on GDP (11%, see [1]).

With the exception of Luxembourg, Switzerland has the highest proportion of foreign population in Europe --almost 2'000'000 persons in 2014, corresponding to 24.3% of the total population. The need for labour to meet development objectives, starting in the early part of the 20^th^ century, coupled with a very restrictive naturalization policy [2], has generated a resident population of foreign citizens --which often includes second and third generation migrants --. In 2014, according to the Federal Office of Statistics, 19% of the total foreigners resident in Switzerland (about 377’000 persons) were born in the country, and almost 540’000 of those born abroad were established in Switzerland since 15 years and more. In 2010, 27% of births were of foreign children [3]. Being a migrant (the terms migrant and foreigner are used interchangeably in the manuscript) carries a risk of higher infant mortality in many receiving countries, but not in all. Actually, in the most open and inclusive societies the effect of low social class, to which many migrants belong, on infant mortality is countered by a high level of attention to specific needs of migrant communities, which in turn brings infant mortality of migrants to the same level of that of the native population [4]. In contrast, in other receiving societies, including Switzerland, foreign communities have a worse reproductive health as compared to the one of the native population. This is possibly explained by a latent attitude of the society towards foreigners, which stresses differences and does not see a poorer health status as a problematic aspect needing intervention [5, 6]. Switzerland, in spite of the high percentage of foreign birth, almost 30% already in 1969 from Italy, Spain and Germany, has until recently paid scanty attention to the analysis of infant mortality of the foreign population, both in official reports and scientific studies, thus contributing to the low visibility of the phenomenon [7, 8]. Although since 2008 an increasing number of publications have targeted specific health needs of migrant communities [9], at the national level only vital statistics, with inherently limited explanatory power, are available to describe infant mortality in the foreign population. A straightforward analysis of these data has not been performed so far. In order to explore whether the high infant mortality rate in Switzerland may be explained by a differential mortality between Swiss and foreign populations, the present paper investigates the rate of infant and neonatal mortality of Swiss and foreign children over the last 30 years.

## Methods

Infant mortality rates (IMR) were computed by dividing the number of deaths occurring before the first anniversary by the number of live births, according to the year of birth. Neonatal mortality rates (NMR) referring to the deaths occurring during the 4 first weeks after birth were also computed by dividing the number of those deaths by the number of live births. Vital statistics from the period 1980 to 2011 were used to compute IMR and NMR s according to year and/or citizenship. The child citizenship at birth and at death respectively was used, from both birth and death registers.

The main analyses were made according to citizenship, contrasting Swiss versus foreigners as a single category. In some instances, the latter was also split into four sub-categories which were defined by grouping the country of origin: 1) Southern European migrants (Spain, Portugal, Italy and Greece); 2) other countries of European Union 15 states and European Free Trade Agreement (EU15/EFTA); 3) Former Yugoslavia (Bosnia and Herzegovina, Macedonia, Kosovo, Serbia, Montenegro, Croatia) and Turkey; and 4) Other countries. This typology helps identifying different migratory flows, which may have a bearing on infant mortality: migrants from Southern Europe actually constituted a traditional migratory flow to Switzerland, recently accelerated by the financial crisis, while migrants from the Balkans arrived since the 1990s, often fleeing conflicts in their country of origin.

Comparisons between groups were done using relative risks, obtained by dividing the IMR for each group with the Swiss one. A relative risk of 1 (or 100%) means the same level of risk for both groups, as values higher than 1 signify excess mortality (1.18 or 118% means for instance 18% of excess risk among foreigners as compared to Swiss citizens). The relative risks are based on the entire population, therefore statistical tests were not computed. Relative risks are unadjusted for possible confounding factors such as maternal age and parity.

## Results

Between 1980 and 2010 more than 2.4 million live births were observed, and 13,601 infant deaths (Table 1). Overall, foreign children represented 23% of live births, 24% of infant deaths and 26% of neonatal deaths respectively.

**Table 1:** Live births, infant deaths and neonatal deaths according to child citizenship, from 1980 to 2010

	Live births			Infant deaths (up to 1 year)				Neonatal deaths (0–27 days)			
	Swiss children	Foreign children	Total	Swiss children	Foreign children	Total	IMR (per 1000) – Total	Swiss children	Foreign children	Total	NMR (per 1000) - Total
1980–89	635297	124453	759750	4600	1028	5628	7.4	2794	701	3495	4.6
1990–93	264810	75999	340809	1550	541	2091	6.1	880	367	1247	3.7
1994–97	243748	85051	328799	1143	451	1594	4.8	771	325	1096	3.3
1998–01	225256	84086	309342	1053	408	1461	4.7	779	290	1069	3.5
2002–05	213118	82070	295188	868	397	1265	4.3	660	303	963	3.3
2006–10	284952	100759	385711	1101	461	1562	4.0	857	391	1248	3.2
Total	1867181	552418	2419599	10315	3286	13601	5.6	6741	2377	9118	3.8

Sources: Vital statistics, birth register and death register

**Fig. 1:**
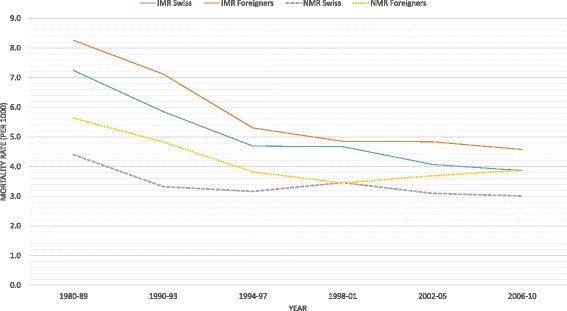
Infant mortality rate (IMR) and Neonatal mortality rate (NMR) 1980–2010 according to the child citizenship. Sources: Vital statistics, birth register and death register

During the 30-years period examined IMR and NMR declined for both Swiss and foreign children, with different shapes but with a consistent gap between the two groups (Fig. 1). In 1980–1989, IMR was 14% higher among foreign children as compared to Swiss children (8.3 per 1,000 and 7.2 per 1,000 respectively), and NMR 28% higher (4.4 per 1,000 and 5.6 per 1,000). In 2006–2010, IMR among foreign children was 18% higher than among Swiss children, and NMR 29% higher (see Table 1). Over the period considered, infant mortality among foreigners was systematically higher, with the highest gap observed during the period 1990–1993 (+21%). At the end of the 1990s there was a converging trend (3% excess infant mortality among foreigners and almost the same level of neonatal mortality). However, between 1998–01 and 2002–05 IMR rapidly declined among Swiss babies, while it remained at the same level among foreigners, again increasing the gap. Over the same period, NMR increased among foreign babies (from 3.4 to 3.9 deaths per 1000 live births) while it declined (from 3.5 to 3.1 deaths per 1000 live births) among Swiss babies.

Table 2 presents the IMR, NMR and relative risk according to country/region of origin. During the period under study, citizens from the Balkan countries as well as other countries of origin (i.e. non UE15/EFTA countries) presented a systematically higher level of IMR than Swiss citizens, reaching 28% of risk between 2002 and 2010. NMR was also higher over the same period, with a risk of 37% for children of Balkan origin. Southern Europeans showed a slightly higher NMR during the last time interval (2006-2010). For the children of the industrialized countries of Europe (EU15/EFTA except Southern Europe), IMR was between 3% and 13% higher than the one of Swiss children during the last two decades of the 20^th^ Century, but similar at the beginning of the 21^st^ Century (2002–2005). Thereafter, in 2006–2010, relative risk was 17% higher, an excess to which largely contributed German, French, and Austrian children.

**Table 2:** Infant and neonatal mortality rates and relative risks according to the region of origin

	Infant mortality rate					Neonatal mortality rate				
	Swiss children	Southern Europe	UE/EFTA	Former Yugoslavia and Turkey	Other	Swiss children	Southern Europe	UE15/EFTA	Former Yugoslavia and Turkey	Other
1980–89	7.2	7.5	7.8	9.5	9.2	4.4	5.3	5.0	6.5	5.9
1990–93	5.9	6.3	6.6	8.2	7.0	3.3	4.4	4.0	5.4	4.9
1994–97	4.7	4.7	4.8	5.5	6.4	3.2	3.4	3.3	3.9	4.8
1998–01	4.7	4.6	5.0	4.9	5.0	3.5	3.7	3.8	3.2	3.5
2002–05	4.1	4.4	4.1	5.2	5.2	3.1	3.4	2.8	4.2	3.7
2006–10	3.9	3.7	4.5	5.0	5.1	3.0	3.3	3.6	4.2	4.5
R.R.(%)										
1980–89	100	103	108	131	127	100	120	113	147	135
1990–93	100	108	113	140	119	100	134	119	163	147
1994–97	100	100	103	118	136	100	109	105	123	153
1998–01	100	99	107	105	107	100	106	111	92	102
2002–05	100	107	100	128	128	100	109	91	136	119
2006–10	100	96	117	128	132	100	109	119	138	148

Sources: Vital statistics, birth register and death register

The limited number of deaths hampers any precise analysis of risk among the smaller foreign communities. Limiting ourselves to the main groups of foreigners (defined as groups observing at least 10 infant deaths among children born during the period 2008–2010), we can however point out the systematic higher level of IMR among foreign citizens compared to Swiss ones (Table 3). This high level especially concerns Turkish and Spanish children (among which the risk of infant death is multiplied by 2.8 and 2.2 respectively). From a transnational comparison perspective, in 2008–2010 migrants from Germany, Portugal, Turkey, Italia, France, and Spain present an increased risk after migration: IMR in Switzerland for those groups is higher than the IMR observed in the country or origin, only Macedonia observing a decreased risk (Table 3).

**Table 3:** IMR in Switzerland and in the country of origin and relative risk as compared to Swiss, 2008–2010

Citizenship of children	Births	Deaths	IMR	R.R. (%) compared to Swiss	IMR in the country of origin
Switzerland	174630	650	3.7	…	…
Germany	7723	44	5.7	153.1	3.6
Portugal	8446	37	4.4	117.7	3.1
Turkey	2925	31	10.6	284.7	7.7
Italia	5525	22	4.0	107.0	3.4
Macedonia	3679	16	4.3	116.8	9.3
France	3076	15	4.9	131.0	3.5
Kosovo	2982	14	4.7	126.1	n.a.
Spain	1374	11	8.0	215.1	3.2

*n.a* not available

Sources: Vital statistics, birth register and death register. IMR in the own countries: OECD 2013 and United Nations 2012 (for Macedonia)

## Discussion

In this paper infant mortality and neonatal mortality rates are used as indicators of the poor health of children of foreign communities in Switzerland. Before discussing the results, it should be stressed that nowadays infant deaths occur rarely. Therefore, those indicators imperfectly translate all potential dimensions of poor pregnancy outcome for foreign children. In particular, differences in children morbidity have been documented between Swiss and non-Swiss groups, and have been shown to persist up to adolescence [10–12].

IMR and NMR are computed using two registers – register of births and register of deaths – that are not linked to each other at an individual level. Therefore, taking into account potential confounding factors, such as parity or age of the mother, is not possible: these variables are only available in the birth register, but not in the death register. This limits the analysis to a strictly demographic approach based on rough rates. However, a previous analysis showed that differences between Swiss and foreigners regarding the parity and age of the mother are rather small and cannot explain all the differences of risks between groups [8].

The position of Switzerland in the world ranking of IMR has progressively worsened during the last twenty years, from the 6^th^ position in 1990 to the 19^th^ in 2010 [13], producing the paradox of a high level of health for the whole population and poor outcomes for the youngest (and the weakest) members of society, the children. A limitation of our data for international comparisons is that the definition of foreign status is based upon the citizenship of the child, because mother’s date of birth allowing linkage between the birth and death registries is no longer available in the latter since 1999 (for a discussion of the implications see [8]). In 93% of the cases child and mothers citizenships coincide. They differ when a foreign mother is married to a Swiss father or when an unmarried foreign mother gives birth to a child that is officially recognized by a Swiss father. In case of binational partners or naturalization of parents prior to birth, the child is classified in the Swiss group. As infant deaths generally occur shortly after the birth (currently, more than 70% of infant deaths occur during the first week), migratory biases (a birth followed by a death abroad, or a birth registered abroad followed by a death in Switzerland) are expected to be very low. Naturalization biases (a child with foreign citizenship at birth but naturalized before the death) are for the same reason very unlikely. It should be noted that, according to the statistics, the mothers of approximately 18% of Swiss babies are born abroad. It is probably one reason why, even if IMR of foreign babies is higher than the one of Swiss babies, the latter have a relative high IMR as compared for instance, to IMR in Sweden. But this assumption cannot be verified with the data that are available.

Our analysis aimed at improving our understanding of the poor and worsening trend of reproductive health by documenting IMR and NMR among foreign children [14]. The results confirm a higher level of infant mortality among foreign children as compared to Swiss ones, a gap that has increased over the last three decades from 14% to 18%. Over the same period, the gap of neonatal mortality has remained remarkably high and stable, 28% to 29% higher for foreign children in 1980 and 2011 respectively. Both the absolute level and the difference of these indicators are worrisome. It is difficult to explain the difference between Swiss and foreign babies only using nationality, a construct that as we have seen above is blurred, but undoubtedly nationality carries a consistent signal over time.

The high level of IMR and NMR among foreign citizens opens the way to different hypotheses that cannot be confirmed due to the limited explanatory power of available data. A national assessment is impaired by the lack of a specific surveillance system of perinatal and infant health, which could aggregate data collected with different instruments beyond vital statistics, such as surveys, administrative registers and censuses that can provide useful information on parental social status and migration trajectory. *Ad hoc* scientific studies investigating the situation have been scanty [15]. Since 1975, the main national agency funding research has financed only three studies focusing on immigrants’ reproductive health, the first one in 2006. The attention of researchers has privileged the overall decrease of infant mortality over time, or recently the decrease of neonatal mortality. For instance, Junker and Berrut [16] observed that during the last 20 years, the mortality rate among children aged 1 to 27 days has halved, although a growing mortality rate was observed in infants in the first 24 h after births, especially among extremely premature births. Therefore, the authors attribute the Swiss situation to an increase in high-risk deliveries (twins and low birth weight and of births among women aged 35 and more). However, other countries are concerned with higher proportions of twins and late births [3] and perform better than Switzerland in terms of pregnancy outcomes.

Among the most common causes of infant mortality of migrants are low social class, inadequate quality of care including poor communication, and stress and discrimination experienced by migrant mothers in receiving societies, particularly but not only newcomers. Social assistance and unemployment statistics confirm the high level of poverty of some groups of foreigners. More than 15% of Turkish in Switzerland were concerned in 2011 by social (cantonal) assistance, which is assigned to families whose income does not allow a decent living. Eight percent of former Yugoslavs and of 4% of Spanish (which experience an increase in poverty) also receive social assistance, in contrast with 2% of Swiss citizens. Data documenting the phenomenon of extreme poverty are not available in Switzerland. However, it is expected that the increase in poverty in Southern Europe during recent years brought precarious groups of migrants in immigration countries, including Switzerland.

Inadequate quality of antenatal care, including poor communication with health care providers, late booking visit and insufficient resources to meet specific needs have been documented in Switzerland [10, 17–20]. Indeed, countries with a low integration of migrants are also those experiencing a higher gap in pregnancy outcome between migrants and natives. This is probably explained by the lack of specific policies, including social and health policy, to respond to specific needs of migrant communities [4]. These policies are considered unnecessary, the common wisdom being that migrants should adapt to the receiving society, and not vice versa. For example, in spite of remarkable and generous efforts to overcome linguistic barriers organized at the local level, Switzerland does not have a national mechanism to ensure translation when doctor and patient do not speak the same language, a not uncommon situation.

The gap of infant and neonatal mortality may be partially explained by the volume of immigration in specific periods, possibly posing a difficult challenge to the health system, which has to deal with newcomers with a high level of stress. Immigration was important during the period 1990–93 with the arrival of refugees from former Yugoslavia (127’500 immigrants every year on average) but low during the periods 1994–97 and 1998–01 (about 85’000 immigrants every year), when the gap among Swiss and foreigners almost closed. Immigration flows increased considerably again during 2002–2007 (100’900) and 2008–2010 (145’800). It has to be noted, however, that even in recent years foreigners from neighboring European countries, who did not experience war or displacement, had a worse infant mortality in Switzerland as compared not only to native Swiss, but also to citizens of their respective country of origin. A particular case is the one of the Turkish population, which has a poor reproductive health in Switzerland as in Germany [21, 22] confirming the similarity between the two countries in terms of integration of the Turkish community.

## Conclusions

Whatever the mechanism, or combination of mechanisms, which cause the observed difference of infant and neonatal mortality between Swiss and foreign populations, no progress has been made over three decades. Critical causal aspects have not been clarified, controlled studies on effectiveness of interventions have not been conducted, and information necessary to define specific policies is still lacking [23]. This situation, which causes a fundamental inequity within the Swiss society towards populations which are an integral and essential part of the social fabric, needs to be challenged by a common, concerted effort bringing together foreign communities, policy makers and clinicians in defining strategies and priorities.

In terms of policy implications, the higher level of mortality among immigrant children raises questions on the capacity of a society to take into account all the specificities of migrant populations. A previous study pointed out the fact that migrant groups have specific needs regarding for instance communication with practitioners or preventive information at the time of pregnancy and after childbirth [4]. With the increasing diversity of migratory flows, in terms of origin, motives for imigration and migrants’ economic and health outcomes, the reproductive health of foreigners becomes a key issue for public health. One of the main targets of Swiss health policies is to guarantee equity in terms of risks between migrants and Swiss citizens, a principle that is enshrined in the Swiss Constitution. Our results demonstrate that the challenge of providing for the same level of risk for all groups is far from been reached, especially when new flows emerge.

Due to the limitations of the data, results presented in this paper are unadjusted and provide no information regarding the factors explaining the risks differentials. Therefore, specific policy measures cannot be suggested. Further analyses are necessary to identify priorities and strategies to reduce observed differentials in risks.

## Abbreviations

EFTA: European free trade agreement
EU15: European Union 15 States
GDP: Gross domestic product
IMR: Infant mortality rate
NMR: Neonatal mortality rate

## References

[1] OECD (2013). Health at a glance 2013. OECD Indicators.

[2] Wanner P, Steiner I (2002). La naturalisation en Suisse. Evolution 1992-2010.

[3] EURO-PERISTAT Project with SCPE and EUROCAT. European Perinatal Health Report. The health and care of pregnant women and babies in Europe in 2010; 2013. www.europeristat.com/images/European%20Perinatal%20Health%20Report_2010.pdf.

[4] Bollini P, Pampallona S, Wanner P, Kupelnick B (2009). Pregnancy outcome of migrant women and integration policy: A systematic review of the international literature. Soc Sci Med.

[5] Todd E (1997). Le destin des immigrés. Assimilation et ségrégation dans les démocraties occidentales.

[6] Lehmann P, Mamboury C, Minder CE (1990). Health and social inequities in Switzerland. Soc Sci Med.

[7] Balthasar H, Spencer B, Addor V (2002). Indicateurs de santé sexuelle et reproductive en Suisse.

[8] Bollini P, Fall S, Wanner P (2010). Vers un système intégré d’indicateurs de la santé maternelle et infantile auprès des collectivités d’origine étrangère en Suisse.

[9] Merten S, Gari S (2013). Die reproduktive Gesundheit der Migrationsbevölkerung. Eine Zusammenfassung der Literatur 2006-2012 Im Auftrag des Nationalen Programms Migration und Gesundheit 2008-2013.

[10] Poretti A, Anheier T, Zimmermann R, Boltshauser E (2008). Neural tube defects in Switzerland from 2001 to 2007: are periconceptual folic acid recommendations being followed?. Swiss Med Wkly.

[11] Merten S, Wyss C, Ackermann-Liebrich U (2007). Caesarean sections and breastfeeding initiation among migrants in Switzerland. Int J Public Health.

[12] Jaeger FN, Hossain M, Kiss L, Zimmerman C (2012). The health of migrant children in Switzerland. Int J Public Health.

[13] United Nations World Population Prospects (2012). The 2012 Revision.

[14] SFSO – Swiss Federal Statistical Office. Vital statistics tables. 2012. https://www.bfs.admin.ch/bfs/fr/home/statistiques/population/naissances-deces.html. Consulted 19 May 2017.

[15] Swiss National Science Foundation. Online database, Projects People Publications P^3^, keywords "reproductive health migrants". 2014. http://p3.snf.ch. Accessed 9 July 2014.

[16] Junker C, Berrut S (2011). Infant mortality in Switzerland. J Epidemiol Community Health..

[17] Bollini P, Stotzer U, Wanner P (2006). Pregnancy outcomes and migration in Switzerland: results from a focus group study. Int J Public Health.

[18] Wolff H, Epiney M, Lourenco AP, Costanza MC, Delieutraz-Marchand J, Andreoli N (2008). Undocumented migrants lack access to pregnancy care and prevention. BMC Public Health.

[19] Kurth E, Jaeger FN, Zemp E, Tschudin S, Bischoff A (2010). Reproductive health care for asylum-seeking women. A challenge for health professionals. BMC Public Health.

[20] Tschudin S, Huang D, Mor-Gültekin H, Alder J, Bitzer J, Tercanli S (2011). Prenatal counseling--implications of the cultural background of pregnant women on information processing, emotional response and acceptance. Ultraschall in der Medizin..

[21] Reeske A, Kutschmann M, Razum O, Spallek J (2011). Stillbirth differences according to regions of origin: an analysis of the German perinatal database, 2004-2007. BMC Pregnancy and Stillbirth.

[22] Spallek J, Lehnhardt J, Reeske A, Razum O, David M (2014). Perinatal outcomes of immigrant women of Turkish, Middle eastern amd North African origin in Berlin, Germany: a comparison of two time periods. Arch Gynaecol Obstet.

[23] Davey Smith G, Krieger N (2008). Tackling health inequities. BMJ..

